# Evaluating ranitidine, pantoprazole and placebo on gastric pH in elective surgery

**DOI:** 10.4103/1658-354X.76508

**Published:** 2011

**Authors:** Tapas Bhattacharyya, Debabrata Sarbapalli, Ranabir Pal, Ujjal Sarkar, Sumit Kar, Kanak Kanti Kundu, Forhad Akhtar Zaman

**Affiliations:** *Department of Anaesthesiology, Institute of Postgraduate Medical Education & Research (IPGME&R), Kolkata, India*; 1 *Department of Anaesthesiology, Burdwan Medical College, Burdwan, India*; 2 *Department of Community Medicine, Sikkim Manipal Institute of Medical Sciences and Central Referral Hospital, Gangtok, Sikkim, India*

**Keywords:** *Pantoprazole*, *preoperative*, *ranitidine*

## Abstract

**Background and Aim:**

Concern about the grim nature of postoperative acid aspiration syndrome grew among the anesthesiologist over the years warranting the need for pre-emptive intervention. The aim of the study is to compare the effects of preoperative oral ranitidine versus pantoprazole given in regulating gastric pH in elective surgery.

**Methods:**

This prospective, parallel group, controlled, randomized, single-blind study was conducted at a tertiary care postgraduate teaching institute at Kolkata, involving 120 participants of either sex, aged 18-60 years of American Society of Anesthesiologists physical status I and II, who were scheduled for elective surgery under general anesthesia lasting for more than 2 h. The participants were divided into three groups. In group A (n=40) participants received placebo tablet, in group B (n=40) participants received ranitidine tablet while in group C (n=40), participants received pantoprazole tablet and their gastric pH estimated serially.

**Results:**

The participants in the three groups were comparable in terms of age, sex, body weight, duration of surgery and type of surgery distribution. In regard to changes in gastric pH trends, there was no statistically significant difference between serial pH values in group A (Friedman test; *P*>0.05) and group C participants. (*P*>0.05). However, the mean preoperative gastric pH values (7.140±.7652) were significantly lower than mean pH values (7.253±.7514) after 2 h postoperatively in group B participants (*P*<0.05).

**Conclusion:**

From the observations and analyses of the present study, it can be inferred that ranitidine is more effective than pantoprazole to raise the gastric pH for prevention of aspiration pneumonitis.

## INTRODUCTION

The condition of aspiration pneumonitis and its dreaded sequelae are well known to the anesthesiologists. And though it is not absolutely preventable, but by adopting some precautions or preventive measures, the chance of aspiration or if it occurs, its sequelae can be brought down to an absolute minimum. Ever since the historic documentation by Mendelson, acid aspiration syndrome, has been a major concern for every anesthesiologist because of morbidity and mortality associated with it.[1–3] Its severity is a function of both the pH and the volume of the gastric juice aspirated. Use of H_2_ inhibitors and/or proton pump inhibitors (PPI) may be useful in high-risk patients (obese, diabetics, American Society of Anesthesiologists (ASA) physical status IV and V, emergency procedures, inadequate starvation, pregnancy, ileus, esophageal dysfunction or surgery).[4]

Its severity depends on both the pH and the volume of the gastric juice aspirated. Studies in rats suggest that in the event of aspiration, the severity of the ensuing pneumonitis depends to a greater extent on gastric fluid pH than on volume.[5]

Gastric acidity may be at its greatest peak following overnight fasting and later in the day when the patient comes to the operation theatre for elective surgery, the greater is the likelihood of acidic juice being present in the stomach, due to anxiety, and hence greater the danger of acid aspiration. Gastric acid volume more than 25 ml and pH less than 2.5 is more important risk factor than gastric volume for pulmonary damage in acid aspiration pneumonitis and have been used as guidelines predicting greater risk of aspiration. Some studies have suggested that a pH of more than 3.5 may also be associated with increased risk of pulmonary damage.[6–9]

PPI have been extensively used for healing of peptic ulcers and found to be superior to H_2_ receptor blockers. Keeping in mind the improved efficacy of PPIs in modifying pH and reducing the gastric acid secretions along with availability of limited data on its usefulness under anesthesia, the present study was planned to evaluate and compare the effect of preoperative oral pantoprazole with commonly used drug ranitidine. The PPIs have been extensively used for healing of peptic ulcers and found to be superior to H_2_ blockers. Pantoprazole is chemically more stable than other PPI compounds under less acidic conditions. Pantoprazole, a substituted benzimidazole derivative, is an irreversible PPI and has been shown to effectively reduce gastric acid secretion. It also provides earlier healing and superior pain relief in peptic ulcer and gastro esophageal reflux disease compared with omeprazole or H_2_ receptor antagonists.[10,11] These agents show higher efficacy in increasing the intragastric pH and suppressing the gastric acid volume with prolonged duration of action, up to 24 hours following a single dose.[12,13] PPIs have been advocated for preanesthetic use to reduce the volume and acidity of any clinically important aspiration.[14]

Awareness regarding the serious nature of acid aspiration syndrome grew among the anesthesiologist and several eminent workers carried out extensive studies on various methods of prevention and also of treatment of established cases. Since there were alarmingly high rate of mortality of established cases despite extensive treatment, the preventive aspect of acid aspiration syndrome were much highlighted and various pioneer workers have worked out several dose regimens and schedules with different therapeutic agents preoperatively, all with a view to prevent or bring down the risk or sequelae of aspiration pneumonitis to an absolute minimum. ASA task force guidelines on preoperative fasting do not recommend routine preoperative use of gastric acid secretion blockers (H_2_ receptor antagonists or PPI’s) or combinations of these and other drugs (antacids, antiemetics or gastrointestinal stimulants) to decrease the risk of acid aspiration syndrome, in otherwise healthy patients scheduled for elective surgical procedures.[15]

Many pharmacological agents have been used for prevention of acid aspiration syndrome but H_2_ receptor blockers are most commonly used. Considering the improved efficacy of PPIs in increasing pH and reducing the gastric acid secretions and the presence of limited data on its perioperative usefulness, we intended to compare the effects of preoperative oral ranitidine and pantoprazole on perioperative gastric pH. To establish the best possible logical solution in Indian socio-political scenario, keeping in the view the fast changes occurring all over the world including South-East Asia this study was undertaken to compare the effects of preoperative oral ranitidine and pantoprazole given at bedtime on the day preceding operation and in the early morning on the day of operation with the control.[16–18] We focused our attention to a comparative study with H_2_ receptor antagonist, PPI and placebo in an attempt to evaluate the efficacy of these therapeutic agents.

## METHODS

The study was approved by the Institutional Ethics Committee. The study was conducted at a tertiary care postgraduate teaching institute at Kolkata from November 2008 to September 2009. One twenty participants of either sex, aged 18-60 years of ASA physical status I and II, without any other additional risk factors for aspiration were included in this study. They were scheduled for elective surgery under general anesthesia lasting for more than 2 h. Exclusion criteria were those with known peptic ulcer disease, participants taking medication known to affect gastric fluid composition or gastric emptying i.e., on PPIs, H_2_ blockers or antacids, ex-smokers, alcohol addiction, pregnant women and those with history of hyperacidity.

### Study design

All participants were randomly assigned into one of three groups. In group A (n=40), participants received placebo tablet, in group B (n=40), participants received ranitidine tablet while in group C (n=40), participants received pantoprazole tablet. The study was prospective, parallel group, controlled, randomized and single blinded.

### Preparation of the patient

All participants were examined preoperatively. Informed written consent was obtained from the participants prior to the study during the preanesthetic check-up. Complete preanesthetic evaluation was performed in each patient including detailed history taking, thorough physical examination (taking weight of each patient) and routine preoperative investigations. A thorough history was taken regarding ailment for which the patient was admitted-past illness especially relevant to gastroenterology, cardiovascular, respiratory, hepatic, excretory and endocrine system, past operation if any. If the patient gave any history of operation in past done under general anesthesia, she was asked in detail about preoperative medication, period of fasting preceding surgery or any vomiting perioperatively. On the day preceding the surgery at 9 p.m and second dose at 6 a.m. on the day of surgery, the participants were given the concerned drugs. Group A participants received placebo, group B received ranitidine (150 mg) and group C received pantoprazole (40 mg).

### Anesthetic technique

The participants were received and identified in the O.T., and an intravenous line established and Ringer’s Lactate infused at body temperature. Then a nasogastric tube of 16-G size was inserted under local lignocaine jelly applied before induction. Gastric fluid samples were obtained by gentle aspiration with a 50 ml syringe at that time and in the interval of 1 h during perioperative period. Then 5 c.c. air was injected through the nasogastric tube to send back the residual amount of gastric juice into the stomach that was in the tube. The aspirated sample was quickly transferred to a clear test tube to be analyzed by pH paper and confirmed by pH meter.

All participants were preoxygenated for 3 minutes and administered fentanyl (2 µg/kg) injection. During preoxygenation the lungs were ventilated, taking care to avoid inflation of the stomach. Anesthetic induction was performed with propofol 2 mg/kg I.V and tracheal intubation was facilitated with atracurium 0.5 mg/kg I.V. Anesthesia was maintained with nitrous oxide and oxygen with incremental dosage of fentanyl and atracurium. When 1 h had elapsed after induction, second sample of gastric juice was aspirated advancing the nasogastric tube further so that the tip entered the stomach. The third sample was also drawn 2 h after induction of anesthesia.

### Statistical analysis

The number of participants required in each group was determined using PS - “Power and Sample Size Calculation” software (Version 2.1.30, February 2003). It was calculated that 40 participants would be required per group to detect a difference of mean pH of 0.5 and inter group standard deviation of 1, study power of 85% and probability of type 1 error as 5%. For statistical analysis, raw data was entered into a MS Excel spreadsheet and analyzed by SPSS 11.5 and Statistica 6.0. Categorized data was analyzed using the *χ*^2^ test. Parametric numerical data was analyzed using one-way ANOVA test and nonparametric data was analyzed using Kruskal Wallis test followed by Mann Whitney U-test and Friedman test followed by Wilcoxon’s matched pairs signed rank test as post hoc analysis. All tests were 2-tailed. A value of less than 0.05 was considered statistically significant and less than 0.01 was highly significant.

## RESULTS

This prospective, parallel group, controlled, randomized, single-blind study was conducted at a tertiary care postgraduate teaching institute at Kolkata, involving 120 participants divided into three groups. In group A (n=40), participants received placebo tablet and in group B (n=40) participants received ranitidine tablet while in group C (n=40) participants received pantoprazole tablet. Demographic characteristics of the participants in the three groups were comparable. The body weights among groups were also comparable. There was no statistically significant difference in age (*P*= 0.7918) and body weight (*P*= 0.9837) between the groups by one-way ANOVA test [Table 1].

**Table 1:** Demographic profile and body weight of study participants

Groups	Sex		Age (in years)			Body weight (in kilograms)		
	Male	Female	Mean	Median	Standard deviation	Mean	Median	Standard deviation
A	21	19	36.88	39.50	8.739	49.70	50.50	7.391
B	19	21	36.08	35.00	11.130	49.80	52.00	7.780
C	21	19	37.73	38.50	12.205	50.00	51.00	7.463

Majority of the participants underwent open cholecystectomy accounting to 37.52% in group A, 32.50% in group B, 40% in group C followed by laparoscopic cholecystectomy, exploratory laparotomy and laparoscopic appendicectomy [Table 2].

**Table 2:** Types of surgery performed on study participants

Surgery	Group A n=40 n (%)	Group B n=40 n (%)	Group C n=40 n (%)
Open cholecystectomy	15 (37.52)	13 (32.50)	16 (40.00)
Laparoscopic cholecystectomy	7 (17.51)	8 (20.00)	7 (17.51)
Exploratory laparotomy	6 (15.00)	5 (12.50)	6 (15.00)
Choledocholithotomy	1 (2.50)	2 (5.00)	1 (2.50)
Incisional hernia repair	4 (10.00)	2 (5.00)	2 (5.00)
Laparoscopic appendicectomy	4 (10.00)	5 (12.50)	6 (15.00)
Total thyroidectomy	2 (5.00)	3 (7.50)	1 (2.50)
Superficial parotidectomy	1 (2.50)	2 (5.00)	1 (2.50)

The duration of surgery in different groups were also comparable. There was no statistically significant difference in duration of surgery between the groups (*P*= 0.8388, one-way ANOVA test) [Table 3].

**Table 3:** Duration of surgery (in minutes) in different study groups

Groups	n	Mean	Median	Minimum	Maximum	Range	Standard deviation
A	40	133.28	135.00	120	147	27	6.051
B	40	132.63	131.00	120	147	27	6.739
C	40	133.45	134.00	123	150	27	6.756

Preoperatively, the mean gastric pH in group A (2.503±.5837) was significantly lower than in group B (7.140±.7652) and group C (6.323±.7069). Participants in group B had significantly higher mean gastric PH than group C (*P*<0.001). Similarly, during intraoperative period, the mean gastric PH values in group A (2.480±.4810) was significantly lower than in group B (7.208±.8722) and group C (6.350±6.350); group B participants had significantly higher gastric pH than group C (*P*<0.001). During postoperative period also, the mean gastric pH in group A (2.385±2.385) was significantly lower than in group B (7.253±.7514) and group C (6.415±.6765); group B participants had significantly higher mean gastric pH than group C (*P*<0.001). In regard to changes to gastric pH trends, there was no statistically significant difference between serial pH values in group A participants (*P*>0.05). However, the mean preoperative gastric pH values (7.140±.7652) were significantly lower than mean pH values (7.253±.7514) after 2 h postoperatively in group B participants (*P*<0.05). There was no statistically significant difference between serial pH values in group C (*P*>0.05) [Table 4].

**Table 4:** Preoperative, intraoperative and postoperative gastric pH of study participants

Groups	Gastric pH estimation	Mean	Median	Standard deviation
Participants	Preoperative	2.503	2.400	0.5837
receiving	Intraoperative	2.480	2.400	0.4810
placebo	Postoperative	2.385	2.400	0.4023
Participants	Preoperative	7140	7.200	0.7652
receiving	Intraoperative	7.208	7.200	0.8722
ranitidine	Postoperative	7 253	7.300	0.7514
Participants	Preoperative	6.323	6.250	0.7069
receiving	Intraoperative	6.350	6.400	6.350
pantoprazole	Postoperative	6.415	6.350	0.6765

## DISCUSSION

In the present prospective randomized placebo-controlled study, the effects of preoperative oral ranitidine was compared with pantoprazole on gastric pH during perioperative period in a group of 120 patients of either sex, aged 18-60 years of ASA physical status I and II scheduled for elective surgery. They were divided into three groups. Patients in group A (n=40) were given placebo, those in group B (n=40) were given 150 mg of ranitidine tablet, those in group C (n=40) were given 40 mg of pantoprazole. In regard to changes in gastric pH trends, there was no statistically significant difference between serial pH values in group A participants. However, the mean preoperative gastric pH values were significantly lower than mean pH values 2 h postoperatively in group B participants. There was no statistically significant difference between serial pH values in group C.

Dammann and associates, observed that single dose of oral ranitidine increases gastric pH to greater than 7.0 for 7-8 h, whereas a single dose of 150 mg administered orally suppressed basal acid output by 70% and pentagastrin stimulated secretion by 40% in healthy volunteers. In addition, 150 mg ranitidine administered orally three times a day produced gastric pH values of 7.0 for 24 hours with previous values of pH 2.0 or below; an average gastric pH 4.0 was maintained even 12 h after the last dose of ranitidine.[19]

Francis and Kwik, studied oral ranitidine for prophylaxis against Mendelson’s syndrome by double blind study in 36 participants undergoing elective surgery. Eighteen participants were given 150 mg ranitidine nights before and also at the morning of operation. The other 18 participants were treated as control. They noted the following observations. In four of the 18 participants who were given ranitidine no gastric sample could be aspirated. In 13 of 14 participants treated with ranitidine (93%) in whom a gastric juice sample was obtained, the pH was greater than 2.5. This was statistically significant (*P* =0.01) and more frequent than in control participants in whom only seven of 18 participants (34%) had a pH greater than 2.5. The high pH was seen as long as 8.50 hours after the morning dose of ranitidine.[20]

Our findings do not match completely with the study of Memis *et al*., on 90 participants (ASA physical status I and II scheduled for elective surgery) to compare the effect of intravenous pantoprazole and ranitidine for improving preoperative gastric fluid properties. In this study, they have compared single dose of intravenous pantoprazole (40 mg) and ranitidine (50 mg) on gastric pH and volume. They found that IV pantoprazole and ranitidine administered 1 h before surgery is equally effective in reducing gastric acidity and volume significantly compared with placebo. This difference may be due to different route of administration of the drug and number of doses given.[21]

Escolano *et al*. showed in their study that a single oral dose of omeprazole, ranitidine or famotidine, given 2-4 h before anesthetic induction, produced a significant increase in gastric pH and a decrease in gastric fluid volume, compared with placebo. There was no significant difference in gastric volume when omeprazole was compared with ranitidine and famotidine, but ranitidine and famotidine produced a significantly greater increase in gastric pH compared with omeprazole.[22] As omeprazole and pantoprazole are both PPIs, so this study corroborates with our present study.

Dehradun (India) study has compared the effects of intravenous administration of metoclopramide, ranitidine and pantoprazole on gastric PH and volume in a double-blind study on 80 females undergoing cesarean section. The researchers concluded that use of pantoprazole was associated with most appreciable changes in characteristic of gastric content and is most effective for decreasing chances of Mendelson syndrome.[23]

Chandigarh (India) study compared the effect of intravenous pantoprazole and an H_2_ receptor blocker ranitidine on gastric secretions in a prospective, randomized, double-blind fashion in 120 adult patients of ASA physical status I and II undergoing elective surgery. They concluded that both the study drugs are equally effective in controlling the gastric fluid properties and thus minimize the risk of pulmonary aspiration syndrome.[24]

Researchers in this field have varying experience than other, where the studies compared PPIs with ranitidine for improving preoperative gastric pH. They noted that ranitidine was equally effective in changing the gastric fluid properties and thus minimizing the risk of aspiration pneumonitis. The improvement in the gastric fluid properties by single dose of pantoprazole was not superior to ranitidine because of use of lower effective dose of pantoprazole 40 mg in comparison to 80 mg or higher required for effective control of gastric-acid hypersecretion.[25–28] Our study is different from other similar studies where we used both the drugs ranitidine and pantoprazole in oral form, which increased the compliance in the study participants. Every possible effort was done to equalize the control and the treated groups by eliminating extraneous factors influencing gastric acidity but all the factors were not within our control. One such uncontrollable factor was individual variation in gastric juice or acid output, by the stomach. However, limitations of the current study included the use of otherwise healthy participants and surrogate endpoints (gastric fluid pH and volume); it would have been better if we have used high-risk patients (e.g., obese, diabetics, esophageal dysfunction) and the outcome data (e.g., incidence of aspiration pneumonia). Thus, clinical relevance of the current study may be weak. However, from a viewpoint of efficiency, we believe that the preliminary study seeking the optimal dose and timing of pantoprazole was necessary before final research assessing the usefulness of the drugs in high-risk patients. We must say that mere chemoprophylaxis may induce a false sense of security to the anesthetist.

In conclusion, the purpose of this study was to evaluate ranitidine and pantoprazole in terms of the ability to raise the gastric pH. Both the drugs under study proved to be efficient in raising the gastric pH to well above 2.5. If we consider the lowest critical pH to be 2.5 then both the drugs were effective in raising the pH to safe level but ranitidine increases gastric pH much more than pantoprazole. If we consider the cost and availability of these two drugs, then ranitidine is much cheaper than the other. From the observations and analyses of the present study, it can be inferred that ranitidine is more effective than pantoprazole to raise the gastric pH for prevention of aspiration pneumonitis.
